# Hospital and Institutional News

**Published:** 1919-03-08

**Authors:** 


### HOSPITAL AND INSTITUTIONAL NEWS.

### PIONEERS IN A NEW MOVEMENT.

The Committee of Management of King's College Hospital have recently agreed to the affiliation to the hospital of the Camberwell Division of the County of London Branch of the British Red Cross Society. This affiliation, believed to be the first of the kind, promises to be of mutual advantage, as on the one hand it will afford the members of this division of the British Red Cross Society an opportunity of carrying on to almost the full extent their noble activities of wartime in time of peace, instead of having to demobilise. On the other hand, King's College Hospital will thereby derive very great benefit by the material and valuable assistance which will be given in the systematic after-care of patients carried out in conjunction with the Almoners' Department, in assistance given at the hospital convalescent home in the country, in the making and padding of surgical splints, in making and repairing articles of linen, in providing special attendants, and by the use of ambulance and service, as well as in clerical and other work, as occasions arise and, lastly, but by no means least, in the gift of practically the whole of the equipment of the auxiliary hospital, which the division organised and has maintained for the past four and a half years. At a time when there is so much speculation as to what use may be made of the British Red Cross Society in peacetime, it may be claimed that this affiliation of the Camberwell Division with King's College Hospital opens up and makes clear in an unmistakable manner the way in which this great organisation can carry on—namely, by affiliation with our great hospitals. In such a covenant King's College Hospital and the Camberwell Division of the British Red Cross Society may be considered pioneers.

### KING'S COLLEGE HOSPITAL FINANCES.

The annual report presented to the Court of Governors at their meeting last week showed that the year's expenditure was £93,187, an increase of £12,392 on that of 1917. As an offset to this increase, however, 5 per cent. more beds were occupied. For military patients £48,800 was received, leaving £44,408 to be provided from hospital funds. The income from ordinary sources was £19,873 (£3,400 less than in 1917); legacies £8,400; and donations £10,115. The ultimate deficiency, therefore, was £6,000, and this was borrowed, the debt on the hospital being now £87,368. Naturally the Committee view this recurring deficiency in income to meet expenditure with concern, especially as the approaching surrender of beds by the military authorities will deprive the institution of substantial payments. To carry on with 350 beds will involve not less than £70,000 per annum; and, as already stated, the ordinary income in 1918 was less than £20,000. Mr. Geo. Trundle, of Southwark, has transferred to the Hospital stocks of the nominal amount of £10,000 for the endowment of a ward for male patients, and a ward in the Hambleden Block has been named the George Trundle Ward. An anonymous donor has given £2,000 5 per cent. War Stock for the endowment of a bed and two cots to be named the Hornshaw Bed and Cots.

### ADMIRAL'S PRAISE OF MEDICAL STAFF.

In his glowing description of the naval attack on Zeebrugge and Ostend in the early part of last year, Sir Roger Keyes does not omit to mention the services of the medical staff. "I desire," the Admiral says, "to make a special reference to the praiseworthy manner in which the medical officers and their staff and volunteer helpers devoted their skill and sympathy to those who were wounded in these operations. Fighting at such close quarters the casualties were bound to be numerous and the wounds likely to be severe. Staff Surgeon James McCutcheon, M.B., was the senior medical officer of the force. I trust that the Lords Commissioners will agree with me in thinking that no branch of the naval service surpassed in zeal and ability the efforts of the medical branch to prove itself worthy of the profession and of the occasion." The Admiralty's gratitude to the medical branch has already found expression in a considerable list of honours and promotions based on the recommendations of Vice-Admiral Keyes.

### A LOSS TO SURGERY.

St. Thomas's Hospital has lost one of its most promising young surgeons by the deeply-regretted death, at the aged of thirty-two, of Mr. Stewart Henry Rouquette, M.Ch. (Cantab), F.R.C.S., O.B.E. He was the son of Mr. G. A. Rouquette. From Eastbourne College he went to Cambridge University, where he graduated in the National Science Tripos in 1908. In 1912 he won the Solly Medal at St. Thomas's Hospital. As Resident Assistant Surgeon to that hospital and Consulting Surgeon to Lady Ridley's Hospital for Officers he established a brilliant reputation as a surgeon.

### ONE HUNDRED THOUSAND POUNDS WANTED.

The Nottingham General Hospital is the only voluntary hospital of any size for many miles round. It serves a large industrial centre and receives a large number of mining casualties. The waiting list, as at some of the larger provincial hospitals, is so colossal, a question of thousands rather than hundreds, that any case which is not a casualty or a real urgency has the most meagre chance of obtaining admission. It is a common occurrence to see three or four extra beds in each ward. There was a consensus of opinion at the Governors' Meeting of the Nottingham General Hospital last November that the City and County War Memorial should take the form of an enlargement and reconstruction of the institution, and the monthly board have now recommended an appeal for £100,000

for this purpose. If the County could become thoroughly aware of the grave economic loss to the community through the lack of a sufficient and efficient local hospital service, the sum asked for, large though it is, would, we are confident, be forthcoming.

### BRITISH PSYCHOLOGICAL SOCIETY.

At a Special General Meeting of the British Psychological Society, held in London, it was unanimously resolved that persons interested (instead, as heretofore, engaged) in the various branches of psychology shall be eligible for membership. It was also decided to institute three special Sections of the Society, devoted to the educational, industrial, and medical aspects of psychology, respectively. Particulars may be obtained from the Honorary Secretary of the British Psychological Society, The Psychological Laboratory, University College, W.C. 1.

### LEICESTER PRACTITIONER'S BEQUEST.

The question of a University College for Leicester in association with a University for the East Midlands has been prominently brought forward in recent years—indeed the question of the local War Memorial taking the shape of a College has already been discussed. A further important step was reached at the last meeting of the Town Council, when Alderman North in moving the adoption of the Education Committee's report, intimated that under the will of the late Dr. J. E. M. Finch, late Medical Superintendent of the Borough Mental Hospital, a bequest of £5,000 was made to the Mayor and Corporation towards the establishment of a University or College, provided that such scheme shall have been embarked upon within a period of twelve months. Alderman North further announced that the fund he had raised for disabled warriors now reached £80,000, and in view of the necessity of an appeal for the University or College, he purposed refraining from any further appeal for the Disabled Warriors Fund. A sum of £150,000 to £200,000 was required for the College for endowment and building and Dr. Finch's bequest was a challenge to the living to carry through a scheme which had already been approved by the Education authorities of the County and Borough.

### REMARKS BY A CORONER.

Dr. Waldo, coroner, in the City Court, in his summing-up in a case, told the jury that in his opinion, if there were more beer there would be fewer strikes. There was, he thought, a closer connection between labour unrest, accentuated by the strain incident on the war, and the shortage of sound, fairly priced drink than many people even dreamt of. A great want, just now, was more beer, and beer worthy of the name of our national beverage and, at the same time, procurable at a less inflated figure than at present fixed and demanded for the poor substitute now provided. Moreover, the industrial worker should receive every encouragement to slake his thirst with pure, light, and appetising beer, rather than with spirits, which was so often, in coroners' and other courts, shown to be, when taken in quantity or habitually, a baneful source of crime, degeneracy and death. Further, he believed the scarcity of good beer and light wines not infrequently led also to the substitution for them of deadly drugs, such as morphine, cocaine, and heroin, and the drug habit in place of the alcoholic.

### DRUGS AND NEEDED RESTRICTIONS.

On another occasion, at Southwark, Dr. Waldo said the evidence pointed to the deceased having formerly taken morphine, and latterly, during the past five or six years, whilst under his doctor, he injected himself with heroin up to five injections in twenty-five hours, obtaining the heroin both from his doctor and from a male friend, the name and address he (the coroner) was unable to obtain from the widow of the deceased. Heroin was derived from morphine, an alkaloid of opium. The only restriction on the supply of heroin was that under the Pharmacy Act of 1898, and Poisons and Pharmacy Act of 1908, whereby it came under the 1st Part of the Schedule, under which it could only be purchased or prescribed by a registered chemist but could be repeatedly obtained in quantity and, unlike cocaine, did not come under the more stringent regulations. The Defence of the Realm Regulation, 40B, made it a punishable offence for any one to obtain or possess cocaine on a prescription from a registered chemist unless such prescription was dated and signed by the medical practitioner with his full name, address and qualifications and marked with the words "Not to be Repeated." The total amount of cocaine to be supplied must be specified upon the prescription. Also, cocaine must not be supplied more than once on the same prescription, which must be retained by the chemist, the ingredients entered in a book, together with the name of the patient, for inspection. He (Dr. Waldo) had for many years advocated similar restrictions, in the case, not only of cocaine, but of other dangerous and deadly poisons, such as morphine, heroin, and laudanum. The sooner such poisons were brought in line with cocaine, the better for the safety of the public, and such restrictions should be retained permanently after the war.

### THE PROBLEMS OF MEDICAL SCHOOLS.

St. Mary's Hospital, London, can offer about a dozen Resident appointments, most of which fall vacant every six months. At present recently qualified women and unqualified men students are appointed in about equal numbers. The hospital's *Gazette* would like to see the men's appointments offered to the demobilised on the ground, for one thing, that "the premature holding of office is not good." At a time, it goes on, when the unqualified student should be regarding medicine in all its aspects "his attention is perforce focussed on one branch to the exclusion of the rest, and this tends to incompleteness and a lop-sided view." The *Gazette* puts forward the suggestion that when such

Residents are appointed they should be prepared to resign at a month's notice if a demobilised man be in need of a term of office in the House. Another point made is that the number of house-surgeoncies and physiciancies is limited and, as the year wears on, there will probably be many more former service men than there are opportunities unless the period of each appointment be curtailed. The suggestion is put forward that the term of office be reduced to three months, so that a greater number may be accommodated and afforded the opportunity to "rub up" their knowledge and re-equip themselves in practical work. Other hospitals with medical schools are probably troubled with the same problems that confront St. Mary's. Ventilation of the subjects may bring solutions.

### A MEDICAL MAN'S PUBLIC SERVICE.

FEW medical practitioners can claim a longer record of public service in a town of adoption than Alderman George Clifton of Leicester, who has passed away in his eightieth year. He came to Leicester in 1871, and a few years later was elected a Borough Councillor. He early secured a seat on the Sanitary Committee, and was at one time Chairman of the Isolation Hospital and the Borough Asylum Committee. In 1897 he filled the Mayoral Chair and his year of office was marked by much philanthropic work. At one time he was Master of the Trinity Hospital, President of the Highfields Hospital (an Institution reviewed by Sir Henry Burdett, K.C.B., in 1914), representative of the Soldiers and Sailors Association, and a member of the Leicester War Pensions Committee. Alderman and Mrs. Clifton celebrated their golden wedding in 1913.

### REPORTS FROM THE PROVINCES.

MR. J. MORGAN, the Secretary of the Pontypool and District Hospital, in sending his annual report, complete, with subscribers' lists, etc., in every detail, says that he hopes that he is first this year. He is. Moreover the report is a very attractive one and shows no signs of hurry in preparation. The workpeople of the district served by the hospital, mostly miners, take a keen interest in their hospital and subscribe liberally to it; such contributions in 1918 amounting to £2,764, being a large proportion of the gross income, £4,676. Another complete report that has reached us, in the proof stage, is that of the Chesterfield and North Derbyshire Hospital. In this district also the workmen evidently appreciate their hospital, having increased their weekly contribution during the war and for twelve months afterwards from 1*d.* to 1½*d.* per week, the total from this source in 1918 was £7,614, as against £5,504 in 1917. It will, we are sure, be only a matter of persuasion to make the three-halfpenny rate a permanent one.

### AN OLD PATIENT'S GRATITUDE.

SOME of the most pleasing incidents of hospital work are those which spontaneously arise from the gratitude of patients. An interesting gift of the kind came before the Board of the Leicester Royal Infirmary, when a local patient who received treatment in the year 1871 presented himself with the request that he might be allowed to address the Board. After referring to the skilful attention he received at the hands of a former honorary surgeon, covering a period of two years, to the care of the nurses (whom he likened to "earth's angels"), to the kindness of the sister of the ward, he said he wished to pay a "debt of honour" which he had long felt he owed, and which he was in a position to do, by asking the chairman's acceptance of a Bank of England note for £100. The donor's sentiments were listened to with pleasure by the Board, and he was heartily thanked for his gift, and informed that his name would be submitted to the anniversary meeting of the governors for election to a life governorship, so that he would be kept in touch with the institution which had played an important, if involuntary, part in the material success which had attended his business activities.

### A SECRETARIAL APPOINTMENT.

MR. R. B. CANNINGS has been appointed Secretary of the City of London Lying-In Hospital, City Road, in succession to Mr. E. Lionel Brown. Mr. Cannings has for the last two and a half years been assistant to Mr. T. Glenton-Kerr at the Queen's Hospital for Children, and the training under such a skilled and successful secretary should be of much value to him in his new appointment.

### PORTS AND THE NATION'S HEALTH.

PROFESSOR E. W. HOPE, of Liverpool, read a paper at the Institute of Public Health, Russell Square, London, on this subject. He expressed approval that in the amended draft of the Ministry of Health Bill the question of the health of seamen was included in its provisions, and hoped for Government action of an international nature, whereby all the ports in the world could exchange experiences on vital matters. A central bureau of information was essential and advisable so that all consular bodies could be given up-to-date information. Referring to merchandise, it had been decided to recommend that wool, a difficult type to handle, should be disinfected not on arrival in ports, but at suitable collecting stations in the country of origin under circumstances making spores and contamination more accessible to sterilisation. It was gratifying to learn of the great improvement in the quality of imported food since the inauguration of inspection in 1908, and the author claimed in this connection that probably no more efficient system than our own existed in the world. War conditions, the enormous transport of troops, labourers, and humanity of all kinds, and the importance of organised shipment of foodstuffs had provided invaluable experience to port health authorities, and it was urgent that through this channel international effort should be made to unify all health work and protection of the world from disease.

### NEW HOSPITALS AS WAR MEMORIALS.

ALL over the country new hospitals are being decided on, often as part of local schemes for memorials of the War. Bognor is to have a cottage hospital, thanks to a gift of £7,000 from Mr. James Fleming; Selby Cottage Hospital, Yorkshire, is to have new quarters modelled on the Goole Hospital; Abercarn, near Newport, has been offered £5,000 by Mr. F. Mills for a local hospital; a public meeting has decided to raise £50,000 to build and partly to endow a hospital at Gosport; the scheme for a new infirmary for Doncaster has been generally endorsed. This batch of decisions and proposals shows that the hospital plan secures more support than any other when war memorials are discussed, and further indicates that the public is at last alive to the need for remedying the shortage of hospital beds, and the long waiting lists which have been the chief defect of the voluntary system hitherto.

### AN UNTAPPED RESERVOIR?

The destination of Red Cross balances is deciding itself in favour of local hospitals, but the points remain to be considered. The first is whether proper steps are being taken to give the local hospitals not merely the local balance but also the names and addresses of its subscribers; and the second the suggestion in the big centres that the balances should be pooled to procure a general fund for the hospitals of the district. Though in these centres joint appeals seem to be coming into favour, the money will probably be divided between the different institutions. But the main point is that the Red Cross has received support from many who had failed to respond to hospital appeals, and an attempt should be made to attract their interest to civil hospitals before it is dispersed by time.

### ADDRESSES OF PATIENTS' FRIENDS.

The Lambeth Board of Guardians were lately called upon to investigate a case in which the friends of an old patient who died suddenly were not notified. It appeared that in 1915 they moved to a fresh house, and, so they stated, sent their new address to the officials. In the interval they had frequently visited their friend but had never inquired whether the altered address had been notified. Consequently, when the death took place, a telegram and letter were sent to the only address in the possession of the officers, but, of course, they were returned. Nine days later they were informed of the death by a letter being returned to them through the "Dead Letter Office." No blame could be attached to the Officers, but the guardians have decided that in future, the nurses shall periodically ask the patients if any alterations have been made in the addresses of their relatives or friends.

### BEDFORDSHIRE'S PEACE OFFERING.

It is proposed that Bedfordshire Peace Thankoffering shall take the form of a Convalescent Home in connection with the County Hospital. The scheme has been launched with every prospect of success. There is an enormous demand on the beds of the hospital, of which there is only one per thousand of the population, as against 3.14 per thousand in London. The Board of Management, therefore, is faced with the problem of enlarging the hospital or otherwise relieving the strain. Enlargement is not favoured, as it would involve rebuilding the Nurses' Home. The better way, it is thought, would be the provision of a Convalescent Home, to accommodate twenty patients from the hospital and twenty of the general public. A sum of £20,000 is required as the initial cost reckoned at £500 per bed, and every one connected with the scheme is optimistic that the public of the county will respond "like a shot" to the appeal now made to them.

### THE SWEDISH WAR HOSPITAL.

The visit of the Crown Prince and Princess of Sweden to London coincided with the date of the luncheon given by the president and committee of the Swedish War Hospital on February 20, and these distinguished visitors were able to be present. The Swedish Ambassador, who presided, called attention to the large amount of work for war hospitals done by the Crown Princess in the last few years, and the Crown Prince, in reply, said it was a matter of regret that they had not been able to visit this country when the War Hospital in Paddington Street was in full swing. He had been asked by the Swedish colony in England to thank the British authorities for the assistance they had given in the work of the hospital, which, started in 1916, had done great service to sick and wounded officers. Lord Burnham, speaking later, said that although the attitude of certain people in Sweden during the war had been questionable, yet the great bulk of Swedish sentiment and opinion was essentially friendly to this country.

### ABERDEEN ROYAL INFIRMARY CHANGES.

Dr. Frederick K. Smith, Senior Assistant Surgeon, has been appointed to the office of Surgeon on the Staff of the Aberdeen Royal Infirmary, and Dr. Thomas Fraser, Senior Assistant Physician, has been appointed Physician. These appointments were due to the resignation of Dr. J. Scott Riddell and the death of Dr. A. H. Lister. The vacancy involved by the resignation of Dr. W. Sinclair, the late Superintendent, has been filled by the appointment of Miss Edmondson, the Matron, who has performed the duties during the last three years.

### THIS WEEK'S DRUG MARKET.

With the exception of a few articles, including camphor, peppermint oil, and lanolin, the easier tendency in prices continues, and in some cases is becoming more pronounced. Business, however, shows signs of improvement, and a fair amount of small buying is going on; this is necessary to meet the consumption demand; but buyers are only committing themselves to small purchases, waiting for things to reach a more stable level before committing themselves to large orders. Among the goods which have been marked down are calumba root, orris root, caffeine, nux vomica, atropine sulphate, eserine, podophyllin, antifebrin, aspirin, sulphonal, salicylic acid, sodium salicylate, salol, phenacetin, artificial oil of wintergreen, glycerophosphates, lemon oil, liquorice juice, cocaine, chloral hydrate, benzonaphthol, benzaldehyde, and barbitone.

